# Cranberry Proanthocyanidins Mediate Growth Arrest of Lung Cancer Cells through Modulation of Gene Expression and Rapid Induction of Apoptosis

**DOI:** 10.3390/molecules16032375

**Published:** 2011-03-11

**Authors:** Laura A. Kresty, Amy B. Howell, Maureen Baird

**Affiliations:** 1 Department of Epidemiology and Public Health, University of Miami Miller School of Medicine and Sylvester Cancer Center, Miami, Florida 33136, USA; 2 Marucci Center for Blueberry Cranberry Research, Rutgers University, Chatsworth, New Jersey 08019, USA; E-Mail: ahowell@aesop.rutgers.edu; 3 Department of Pathology, The Ohio State University, Columbus, Ohio 43240, USA; E-Mail: Maureen.baird@osumc.edu

**Keywords:** cranberry, tannins, proanthocyanidins, flavan-3-ols, apoptosis, cancer cells, global gene expression, cell cycle

## Abstract

Cranberries are rich in bioactive constituents purported to enhance immune function, improve urinary tract health, reduce cardiovascular disease and more recently, inhibit cancer in preclinical models. However, identification of the cranberry constituents with the strongest cancer inhibitory potential and the mechanism associated with cancer inhibition by cranberries remains to be elucidated. This study investigated the ability of a proanthocyanidin rich cranberry fraction (PAC) to alter gene expression, induce apoptosis and impact the cell cycle machinery of human NCI-H460 lung cancer cells. Lung cancer is the leading cause of cancer-related deaths in the United States and five year survival rates remain poor at 16%. Thus, assessing potential inhibitors of lung cancer-linked signaling pathways is an active area of investigation.

## 1. Introduction

Lung cancer is the leading cause of cancer related death among men and women in the United States and despite recent advances in treatment overall prognosis remains poor [1]. The development of effective agents for the prevention and treatment of lung cancer is an active area of investigation. Smoking is the principal cause of lung cancer [2]; however, risk increases with exposure to second hand smoke, radon, asbestos, radiation, arsenic, aluminum, chromium, cadmium and select organic chemicals [3]. The potential protective role of diet in lung cancer is still being unraveled. To date, epidemiological studies strongly support that diets rich in fruits may reduce lung cancer risk, as extensively reviewed in the World Cancer Research Fund report [4]. Fruits contain a multitude of bioactive food constituents with pleiotropic health benefits. Cranberries (*Vaccinium macrocarpon* Ait.), for example, reportedly have antimicrobial, antiviral and more recently, anticancer functions [5,6,7,8,9,10,11,12,13,14,15,16,17]. The current study sought to investigate potential cancer inhibitory mechanisms associated with a proanthocyanidin rich cranberry fraction (PAC) in “resistant” lung cancer cells. The investigation of such food-derived agents holds particular promise given the repeated negative results of vitamin/mineral/antioxidant supplementation trials in at risk cohorts [18,19,20,21]. Promising food-derived agents with cancer inhibitory effects supplied at behaviorally achievable levels are likely to be well tolerated and safe. These are important considerations for relatively healthy cohorts who may consume cancer protective agents for extended periods to derive maximum health benefits. Understanding the specific mechanisms of cancer inhibition is important not only for preventive interventions, but may hold promise for reversing a “resistant” phenotype prior to cancer chemotherapy. 

## 2. Results and Discussion

### 2.1. Modulation of global gene expression patterns by PAC

**Figure 1:**
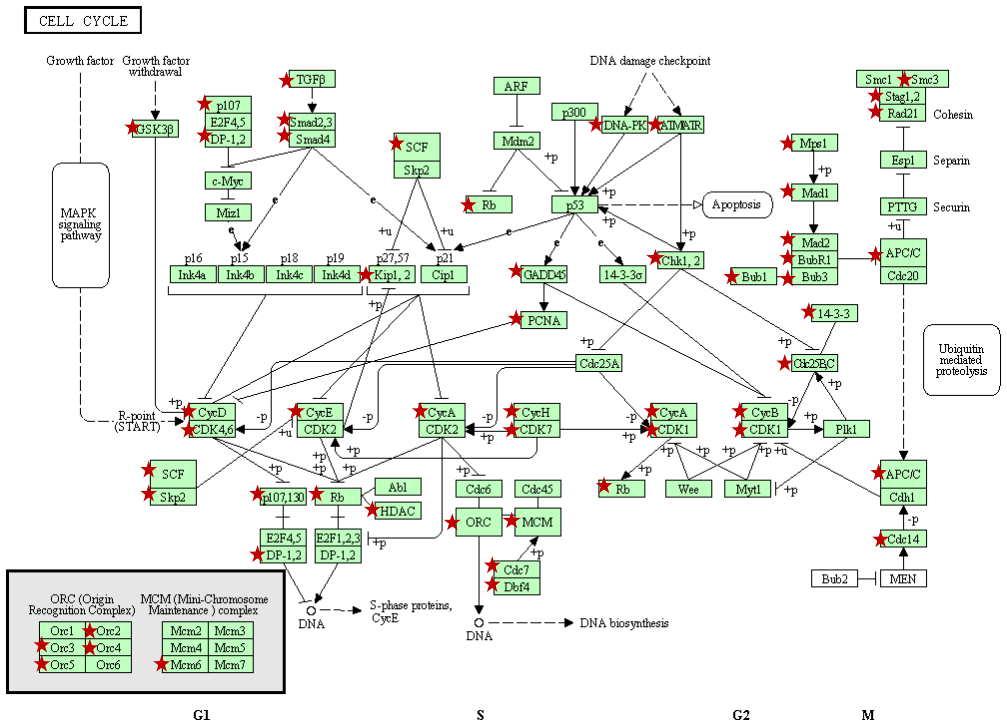
Cell cycle pathway represented in KEGG [24,25]. Red stars mark genes down regulated following PAC treatment of NCI-H460 cells compared to vehicle treated cells based on global gene expression results. As illustrated, PAC alters expression of a large number of genes involved in all phases of cell cycle regulation, including the G1/S and G2/M transitions and DNA replication.

To explore the mechanisms and signaling cascades linked to the cancer inhibitory potential of PAC we utilized global gene expression analysis of NCI-H460 human lung cancer cells which were treated with 50 µg/mL PAC or vehicle for 6 hours. This concentration of PAC was chosen based on earlier work which had determined the IC_50_ to be 50 µg/mL [17]. Ease analysis was employed to investigate the effects of PAC on over-represented Gene Ontology (GO) categories (Table 1). The top 30 biological processes down-or up-regulated greater than 2-fold are displayed in Table 1. As shown a large number of processes linked to cell death were significantly up-regulated by PAC treatment including regulation of apoptosis, regulation of programmed cell death, positive regulation of cell death, positive regulation of apoptosis, and apoptotic mitochondrial changes. The dominate biological processes down-regulated following PAC treatment included metabolism, protein modifications, and cell cycle linked processes as illustrated by down regulation of M phase of mitotic cell cycle, mitosis, M phase, cell cycle checkpoint, and regulation of cell cycle process. Figure 1 illustrates the cell cycle pathway in KEGG with red stars marking down-regulated markers following PAC treatment of NCI-H460 cells compared to vehicle treated NCI-H460 cells. A number of cell cycle related markers were validated utilizing real time PCR and RT² Profiler PCR Arrays including, BCCIP, CCNB1, CCND1, CCNT1, CDC2, CDC16, CDC20, CDK4, CDK6, CDKN3, CHEK2, GTF2H1, HUS1, KNTC1, MAD2L1, MCM5, MKI67, MNAT, MRE11A, PCNA, RAD1, RAD17, SERTAD1, SUMO1 and TFDP1. Further evaluation of signaling pathways utilizing PANTHER further supported the general alterations noted on gene ontology categories. Detoxification processes were up-regulated by PAC treatment, as a number of glutathione *S*-tranferases were significantly increased. PAC treatment also down-regulated DNA metabolism, cell cycle, meiosis, mitosis and interestingly oncogenesis signaling. In addition, analysis of KEGG pathways following PAC treatment revealed effects on erbB and mTor signaling, pathways altered in lung carcinogenesis [22,23]. 

**Table 1:** Over-represented gene ontology categories deregulated in NCI-H460 cells by PAC treatment.

Up-Regulated Biological Processes (n = 30/221)	%	*P*-Value	Down-Regulated Biological Processes (n = 30/207)	%	*P*-Value
Protein transport	5.89	7.2E-11	RNA metabolic process	7.46	9.5E-21
RNA metabolic process	6.81	3.8E-09	Cellular protein metabolic process	15.79	1.4E-19
Cellular protein metabolic process	15.17	7.2E-09	RNA processing	4.61	8.9E-16
RNA processing	4.24	3.5E-08	M phase of mitotic cell cycle	2.21	1.1E-12
Intracellular protein transport	3.02	3.9E-07	Mitosis	2.16	2.1E-12
Golgi vesicle transport	1.27	4.3E-06	M phase	2.91	4.0E-12
RNA splicing	2.29	1.2E-05	DNA repair	2.59	6.5E-12
Cellular protein complex assembly	1.44	2.7E-05	Regulation of gene expression	17.66	1.3E-11
Protein modification process	9.24	5.2E-05	Regulation of macromolecule biosynthetic process	17.51	1.4E-11
Regulation of gene expression, epigenetic	0.80	7.0E-05	mRNA metabolic process	3.13	3.8E-11
Regulation of apoptosis	5.37	1.2E-04	DNA metabolic process	3.96	1.7E-10
ncRNA processing	1.56	1.2E-04	Modification-dependent macromolecule catabolic process	4.37	3.1E-10
mRNA metabolic process	2.73	1.2E-04	mRNA processing	2.74	3.1E-10
phospholipid biosynthetic process	0.97	1.4E-04	Cellular protein catabolic process	4.54	3.9E-10
Regulation of programmed cell death	5.40	1.7E-04	Regulation of cellular biosynthetic process	17.91	4.3E-10
mRNA processing	2.40	1.9E-04	Regulation of nucleobase, nucleoside, nucleotide and nucleic acid metabolic process	17.14	8.0E-10
Positive regulation of cell death	3.09	3.0E-04	Protein catabolic process	4.61	1.6E-09
Negative regulation of macromolecule metabolic process	4.88	3.5E-04	RNA splicing	2.44	2.0E-09
Positive regulation of programmed cell death	3.06	3.7E-04	Regulation of transcription	15.79	1.1E-08
Positive regulation of apoptosis	3.04	4.0E-04	Protein transport	5.25	1.5E-07
Apoptotic mitochondrial changes	0.40	4.3E-04	Protein modification process	9.15	5.5E-07
Glycerophospholipid biosynthetic process	0.68	5.3E-04	Cell cycle checkpoint	0.94	1.1E-06
Negative regulation of cellular metabolic process	4.76	5.6E-04	Regulation of cell cycle process	1.07	6.2E-06
Chromatin modification	2.05	5.9E-04	Negative regulation of macromolecule metabolic process	4.89	7.3E-06
ncRNA metabolic process	1.77	6.3E-04	DNA replication	1.56	1.3E-05
Actin filament organization	0.71	6.6E-04	Negative regulation of nucleobase, nucleoside, nucleotide and nucleic acid metabolic process	3.51	2.7E-05
Induction of programmed cell death	2.31	1.1E-03	RNA biosynthetic process	2.21	2.9E-05
Heme metabolic process	0.31	1.2E-03	Translation	2.42	3.0E-05
Negative regulation of gene expression	3.42	1.2E-03	DNA damage response, signal transduction	0.79	3.1E-05
DNA metabolic process	3.42	1.4E-03	Negative regulation of gene expression	3.43	5.7E-05

### 2.2. Pac induces rapid and significant apoptosis in lung cancer cells

**Figure 2:**
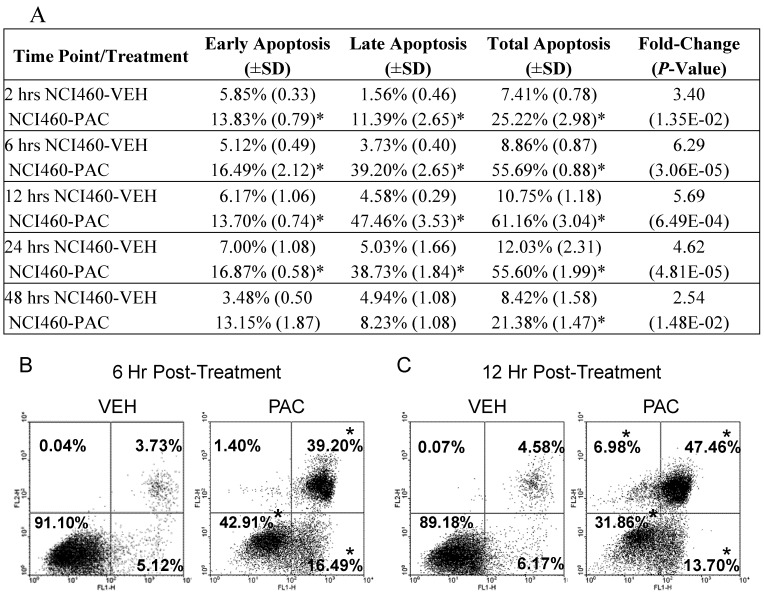
PAC (50 μg/mL) induces cell death in NCI-H460 lung cancer cells. (A) summary of the effects of PAC on early, late and total apoptosis at 2, 6, 12, 24 and 48 hours post-treatment as determined by Annexin V-FITC staining. PAC treatment induced significant early apoptosis (B and C, lower right quadrant), late apoptosis (upper right quadrant) and total apoptosis at 2, 6, 12, and 24 hours. The largest magnitude of apoptosis induction occurred following 6 and 12 hours of treatment as illustrated in (B) and (C). PAC induces significant necrosis (C, upper left quadrant) following 12 and 24 hours of PAC treatment as evidenced by the increase from <1.00% necrosis in vehicle treated NCI-H460 cells to 6.98% in PAC treated cells 12 hours post-exposure. The values represent means ±SD of three independent samples per experimental treatment and time-point (*P* < 0.05, two-tailed *t* test). Asterisks indicate a statistically significant difference between PAC and vehicle treated cells. Reported fold-change values refer to the fold-change induced by PAC treatment compared to vehicle in terms of total apoptosis induction.

Next, PACs cell death inducing effects were further investigated over time in lung cancer cells via Annexin V-FITC staining methods and flow cytometry. As Figure 2 shows, PAC treatment resulted in significant and rapid apoptosis induction, with maximal apoptosis of 6.29-fold occurring 6 hours post-treatment. Total apoptosis occurred at 25.2, 55.7, 61.2, 55.6 and 21.4% at 2, 6, 12 and 24 hours, respectively. Specifically PAC induced early, late and total apoptosis following 2, 6, 12 and 24 hours of PAC treatment; however, by 48 hours there was a significant, but only modest 2.5 fold increase in total apoptosis supporting that the cells are starting to recovery from PACs cell death inducing effects at this late time-point. At 2 hours post-PAC treatment the majority of apoptosis is early, but rapidly shifts to late apoptosis by 6 hours. The data also shows significant reductions in “unstained” or live cells as displayed in the lower left quadrant of Figure 2B and 2C. The reductions in unstained vehicle *versus* PAC treated cells were 18.11, 48.19, 57.32, 48.91 and 13.74% at 2, 6, 12, 24, and 48 hours, respectively supporting that the greatest PAC-induced cell death occurs between 6 and 24 hours. The gene expression results coupled with these findings led us to further explore and validate specific apoptotic markers utilizing real time PCR as described in the methods section. Table 2 summarizes the results of the validated apoptotic markers and their potential functions. 

In summary, PAC treatment resulted in rapid and significant induction of cell death in NCI-H460 lung cancer cells. This line is known to express wild type p53 [26], but also has been documented to over-express X-linked inhibitor of apoptosis protein (XIAP) resulting in suppressed activation of downstream effector caspases and apoptotic resistance [27]. Resistance is problematic in the context of cancer prevention as well as chemotherapy; thus, it is particularly promising that PAC has potent cell death inducing effects in a “resistant” cell line. Furthermore, global gene expression results showed that PAC down-regulated expression of a number of inhibitor of apoptosis proteins (IAPs) including BIRC1, BIRC2, BIRC4 or XIAP, and BIRC6, many of which have been been linked to apoptosis resistance in the presence of anticancer drugs [28,29]. As shown in Table 2, PAC down-regulates a number of additional anti-apoptotic molecules, including BAG4, BNIP2, and BNIP3L. Conversely, the RT² Profiler PCR Apoptosis Array validated a number of pro-apoptotic markers [31,32] as up-regulated following PAC treatment including BID, multiple pro-apoptotic TNF superfamily members and related adapter molecules, as well as p73 [33]. P73 is a p53 family member linked to cell death induction via apoptosis and type II cell death or autophagy [34]. Kim *et al.* reported that up-regulation of autophagy occurred following treatment with inhibitors of caspase-3 and mTOR resulting in enhanced radiosensitivity in a mouse model of lung cancer [35]. PAC treatment of NCI-H460 cell decreased pro-apoptotic, CASP3 [36] which may be linked to activation of type two cell death. PAC treatment up-regulated pro-apoptotic Bcl-2 family members [37] such as Bok, Bax, and Bad; the latter two molecules are known to be inactivated by tobacco specific carcinogens in lung epithelial cells [38,39,40,41]. Although further analysis of specific caspase molecules is warranted, the gene expression results coupled with the real-time PCR validation data support that apoptosis induction is mediated in part by the activation of death receptors belonging to the tumor necrosis factor receptor gene superfamily. 

**Table 2:** Apoptotic associated genes validated by real-time PCR as up- or down-regulated in NCI-H460 cells following PAC treatment.

*Up-Regulated by PAC Treatment* (>2.0 fold)			
Gene	Name	Fold-Change	Function
BCL2L10	BCL2-like 10 (apoptosis facilitator)	+3.25	Anti-apoptotic member of the Bcl-2 family that blocks apoptosis in the mitochondrial death pathway, but not in the death receptor pathway [42].
BID	BH3 interacting domain death agonist	+3.25	Pro-apoptotic member of Bcl-2 proteins and encodes a death agonist that heterodimerizes with either agonist BAX or antagonist BCL2.
DFFA	DNA fragmentation factor	+2.00	A substrate for caspase-3 and triggers DNA fragmentation during apoptosis [43].
MCL1	myeloid cell leukemia sequence 1 (BCL2-related)	+2.00	Involved in the regulation of apoptosis *versus* cell survival and maintenance of viability, but not of proliferation. Two isoforms have been identified, isoform 1 inhibits apoptosis and isoform 2 promotes apoptosis [44].
TNF	tumor necrosis factor	+9.19	Cytokine that binds to TNFRSF1A/TNFR1 and TNFRSF1B/TNFBR, involved in the regulation cell proliferation, differentiation, apoptosis, lipid metabolism, and coagulation. Induces cell death of certain tumor cell lines.
TNFRSF10A	tumor necrosis factor receptor superfamily, member 10a	+3.03	Transduces cell death signal and induces cell apoptosis via activation by tumor necrosis factor-related apoptosis inducing ligand (TNFSF10/TRAIL) [45].
TNFRSF25	tumor necrosis factor receptor superfamily, member 25	+14.93	TNFSF12/APO3L/TWEAK receptor, interacts directly with the TRADD, mediates activation of NF-kappa-B and induces apoptosis [46)].
TNFSF7	tumor necrosis factor receptor superfamily, member 7	+2.83	Cytokine that binds to CD27 and involved in T-cell activation. Induces proliferation of co-stimulated T-cells and enhances the generation of cytolytic T-cells [47].
TP73	tumor protein P73	+22.63	Postulated tumor suppressor protein and p53 family member. Family members include p53, p63, and p73 and have high sequence similarity, which allows p63 and p73 to transactivate p53-responsive genes causing cell cycle arrest and apoptosis. [33].
TRADD	TNFRSF1A-associated via death domain	+12.13	Adaptor molecule that interacts with TNFRSF1A/TNFR1 and mediates programmed cell death signaling and NF-kappaB activation. This protein reduces recruitment of inhibitor-of-apoptosis proteins (IAPs) by TRAF2 [48].
TRAF3	TNF receptor-associated factor 3	+2.43	Adapter protein and signal transducer that links members of the tumor necrosis factor receptor family to signaling pathways. Involved in the activation of NF-kappa-B and JNK and in apoptosis [49].
BAG4	BCL2-associated athanogene 4	-2.46	Member of the BAG1 anti-apoptotic protein family [31,32].
XIAP	baculoviral IAP repeat-containing protein 4	-6.96	Apoptotic suppressor through binding to tumor necrosis factor receptor-associated factors TRAF1 and TRAF2 [28,29].
BFAR	bifunctional apoptosis regulator	-3.25	Apoptosis regulator with bifunctional anti-apoptotic activity for apoptosis triggered by death-receptors and mitochondrial factors [50].
BNIP2	BCL2/adenovirus E1B 19kDa interacting protein 2	-6.50	Member of the BCL2/adenovirus E1B 19 kD-interacting protein family. Its specific function is unknown; however, it interacts with the E1B 19 kD protein which is responsible for the protection of virally-induced cell death, as well as E1B 19 kD-like sequences of BCL2, an apoptotic protector [51].
BNIP3L	BCL2/adenovirus E1B19kDa interacting protein 3-like	-3.25	Same as BNIP2. May also function as a tumor suppressor and inhibits apoptosis induced by BNIP3 [51,52].
CARD8	caspase recruitment domain family, member 8	-3.73	Postulated to be a component of the inflammasome, a protein complex that plays a role in the activation of proinflammatory caspases. Also, acts as an adaptor molecule negatively regulating NFKB activation, CASP1-dependent IL1B secretion, and apoptosis [53].
CASP3	caspase 3, apoptosis-related cysteine peptidase	-8.00	Involved in the activation cascade of caspases responsible for apoptosis execution. An effector caspases, responsible for cleaving downstream substrates [36].
CASP4	caspase 4, apoptosis-related cysteine peptidase	-13.00	An initiator caspase able to cleave and activate its own precursor protein, as well as caspase 1 precursor.
CRADD	CASP2 and RIPK1 domain containing adaptor with death domain	-3.03	Apoptotic adaptor molecule specific for caspase-2 and FASL/TNF receptor-interacting protein RIP [54].
LTBR	lymphotoxin beta receptor, TNFR superfamily member 3	-2.83	Receptor for the heterotrimeric lymphotoxin containing LTA, LTB, and TNFS14/LIGHT. Pro-apoptotic via TRAF3 and TRAF5 [55,56].
TNFS8	tumor necrosis factor (ligand) superfamily, member 8	-3.73	A cytokine that belongs to the tumor necrosis factor (TNF) ligand family and has been reported to induce cell proliferation [57].
TP53BP2	tumor protein p53 binding protein	-5.66	Regulates apoptosis and cell growth through interactions with other p53 regulatory molecules. Inhibits the ability of APPBP1 to conjugate NEDD8 to CUL1 decreasing apoptosis induction by APPBP1. Impedes cell cycle progression at G2/M checkpoint [58,59].

**Figure 3:**
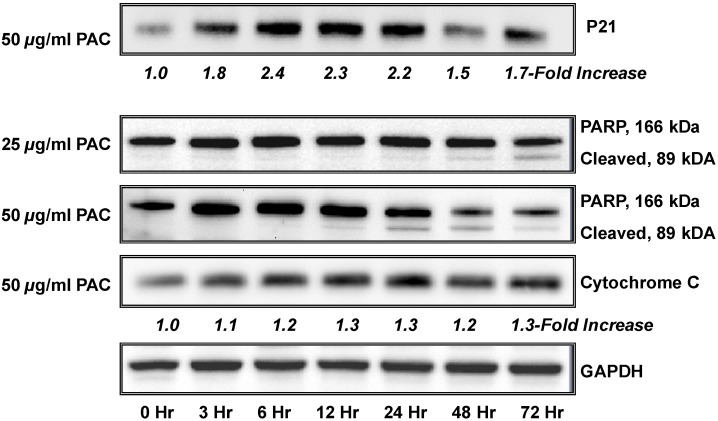
PAC treatment is associated with time-dependent changes in the expression of cell cycle regulatory and apoptotic molecules. NCI-H460 cells (1.0 × 10^5^ cells) were grown for 35 hours, rinsed with PBS, treated with PAC (50 µg/mL) in triplicate, harvested at various time points (0–72 hours), and subjected to immunoblot. Expression values were normalized to the appropriate loading control (GAPDH) and a fold-change from baseline or first detection level was calculated. A time-dependent increases in expression of P21 was noted as early as 3 hours with maximal increased expression at 12–24 hours. A time course of increased expression of the apoptotic markers cytochrome C and PARP are shown. PARP cleavage was both time and dose dependent.

Next, a limited number of specific proteins were analyzed to further verify the effects of PAC on the apoptotic marker PARP1 and P21, an important mediator of cell cycle arrest as illustrated in Figure 3. We have previously reported that PAC decreases S-phase and causes arrest of NCI-H460 cells at the G1 checkpoint; however, we have recently found that PAC can induce arrest at the G2 checkpoint in esophageal adenocarcinoma cells (EAC) (unpublished data) supporting that specific cell death inducing effects differ between cell lines, likely due to the molecular profile of the individual cell line under investigation. P21 is reported to mediate cell cycle arrest in response to the p53 checkpoint [60] and the NCI-H460 cell line expresses wild type p53; whereas p53 is mutated or deleted in our EAC cells. In addition, induced activation of p21 has been linked to lung cancer cell growth inhibition and enhanced chemosensitivity to cisplatin [61]. 

## 3. Experimental

### 3.1. Cell cultures

The lung adenocarcinoma cell line NCI-H460 originated from a non-small cell lung cancer of the large cell type and is available through American Type Culture Collection [62]. Cancer cells were grown in Dulbecco’s modification of Eagle’s medium (DMEM) containing L-glutamine (2.0 mM), penicillin (10^4^ units/mL), sodium pyruvate (1 mM), and FBS (0-10%, depending on the experiment). Cells were maintained as monolayers (37 °C, 5% CO_2, _95% air).

### 3.2. Cranberry proanthocyanidins

Cranberry fruit (*Vaccinium macrocarpon* Ait.) was collected at the Marcucci Center for Blueberry and Cranberry Research, Chatsworth, NJ, USA. The cranberry PAC-rich powder was prepared Dr. Amy Howell (Rutgers University, Chatsworth, NJ) as previously reported [63]. In brief, purified cranberry proanthocyanidin extract was isolated from cranberries of the ‘Early Black’ cultivar using solid-phase chromatography according to well-established methods [63,64,65]. The fruit was homogenized with 70% aqueous acetone, filtered, and the pulp was discarded. Acetone was removed and the cranberry extract was suspended in water, applied to a preconditioned C-18 solid phase chromatography column, and washed with water to remove sugars, followed by acidified aqueous methanol to remove acids. The fats and waxes were retained on the C-18 sorbent. The total polyphenolic fraction containing anthocyanins and flavonol glycosides as well as the proanthocyanidins (confirmed using reverse phase HPLC with diode array detection) was eluted with 100% methanol and dried under reduced pressure. The total polyphenolic fraction was suspended in 50% EtOH and applied to a preconditioned Sephadex LH-20 column, which was washed with 50% EtOH to remove low molecular weight phenolics (anthocyanins and flavonol glycosides). Remaining proanthocyanidins that adsorbed to the LH-20 column were eluted with 70% aqueous acetone. Elution of the proanthocyanidins was monitored using diode array detection at 280 nm. The absence of absorption at 360 and 450 nm confirmed that anthocyanins and flavonol glycosides were successfully removed. Acetone was removed under reduced pressure, and the resulting purified proanthocyanidin extract was freeze-dried. The presence of proanthocyanidins with A-type linkages was confirmed using matrix-assisted laser desorption ionization (MALDI-TOF MS) or electrospray ionization (DI/ESI MS) as previously described [61]. To summarize, current technologies including ^13^C NMR, electrospray mass spectrometry, MALDI-TOF MS, and acid-catalyzed degradation with phloroglucinol have been employed to characterize the profile and concentration of proanthocyanidins present in the extract under evaluation [63,64,65]. As previously reported, the proanthocyanidin molecules consisted of epicatechin units with mainly DP of 4 and 5 containing at least one A-type linkage [64].

### 3.3. Flow cytometry analysis of cellular apoptosis

NCI-H460 cells (1.5 × 10^6 ^cells) were incubated for 24 hours before PAC [0.25 or 50 µg/mL of media] or vehicle (<0.001% ETOH) was added. Apoptosis was evaluated in at least triplicate at each time point which included 2, 6, 12, 24, and 48 hours post-treatment using Annexin V-FITC staining methods and counted using a FACSCalibur flow cytometer with a minimum of 10,000 cells counted [66]. Analysis of apoptosis was performed using WinMDI software (Joseph Trotter; http://pingu.salk.edu.software.html) and ModFit LT software (Verity Software, Topsham, ME, USA).

### 3.4. Western blot analysis

NCI-H460 cells (1.0 × 10^6^) were incubated for 24 hours, rinsed with PBS, treated with PAC (0.25 or 50 µg/mL) or vehicle (<0.001% ETOH) and harvested at 0, 3, 6, 24, 48 and 72 hours post-treatment. Cell lysates were prepared using Cell Signaling lysis buffer. Protein was quantified using the Quick Start Bradford Protein Assay Kit (BioRad) and equivalent protein amounts were loaded into precast NuPage Novex Bis-Tris 10% gels (Invitrogen). Immunoblot was performed using commercially available antibodies from Santa Cruz Biotechnology (Santa Clara, CA, USA) to proteins of interest including P21 (sc-6246), Cytochrome C (sc-13156), PARP1 (sc-8007), and GAPDH (sc-32233) as loading control. Expression values were determined by chemiluminescent immunodetection and normalized to GAPDH. Fold-change from baseline or first detection level was calculated. Positive fold-change values indicate increased expression. 

### 3.5. Isolation of RNA and synthesis of cDNA

Total RNA was prepared from cells using TRIzol reagent (Invitrogen, Carlsbad, CA, USA) and quantity and quality were determined by Nanodrop using the Bioanalyzer 2100 capillary electrophoresis system (Agilent) at the Ohio State University (OSU) Microarray Core. Reverse transcription of purified RNA to cDNA was generated using 1.0 µg of total RNA, oligo(dT), and random hexamers (Applied Biosystems, Foster City, CA, USA) as primers for first strand cDNA synthesis under the following conditions: 30 °C for 5 minutes, 37ºC for 15 minutes, 42 °C for 60 minutes, 50 °C for 10 minutes, and 70 °C for 15 minutes. The first strand synthesis product was treated with RNase H (15 minutes, 37 °C) prior to use in the real-time reaction. 

### 3.6. Microarray studies

NCI-H460 lung cancer cells (2.0 × 10^6^ cells) were treated with vehicle or PAC [50 µg/mL] for 6 hours. One microgram of RNA per condition was reverse-transcribed, labeled by incorporating biotinylated nucleotides during *in vitro* transcription, and hybridized to the human U133 2.0 Plus chip per manufacturer recommendations (Affymetrix, Santa Clara, CA, USA). Specific transcripts bound to the corresponding oligonucleotide probes and the biotinylated cRNA bound fragments were detected using a streptavidin-antibody-phycoerythrin conjugate. 

### 3.7. Validation by Real Time PCR

Real time PCR for differentially expressed genes of interest was performed utilizing RT² Profiler PCR Arrays (SABiosciences; Fredrick, MD, USA) and the iCycler IQ (Bio-Rad) to perform real-time PCR. Specifically, the apoptosis and cell cycle PCR pathway focused ‘Profiler Arrays’ were utilized permitting evaluation of 84 pathway linked probes for gene expression profiling. Relative changes in gene expression were calculated by 2 ^- ∆∆Ct^, where ∆∆Ct = ∆Ct (treated) – ∆Ct (untreated). Data were normalized to expression levels of a combination of control genes including HPRT1, RPL13A and GAPDH.

### 3.8. Statistical and microarray analysis

Results are presented as the mean value ± SD for the apoptosis experiments. Data were evaluated for statistical significance using the Student’s *t* test (two-sided, *p* < 0.05). Microarray data was analyzed utilizing previously described techniques [67,68,69,70,71]. Briefly, analysis was performed utilizing unbiased differential gene expression, comparing relative fluorescence intensities between arrays, and Affymetrix images were transformed into CEL files utilizing GCOS software (Affymetrix). Gene expression levels were estimated from GeneChip probe intensities using the WEDGE++ algorithm [69]. WEDGE++ computes *p*-values based on nonparametric probe-level multi-array chi-square tests for differential gene expression. Next, two-fold differentially expressed genes were analyzed using Expression Analysis Systemic Explorer (Ease software, DAVID 6.7, updated March 2009; http://david.abcc.ncifcrt.gov/) [67,71] to identify over-represented biologic themes classified by gene ontology categories. Signaling pathways were further investigated by viewing the PANTHER (Protein ANalysis THrough Evolutionary Relationships) biological processes which is linked to the DAVID website. PANTHER is a database of phylogenestic trees of protein-coding gene families supporting a number of database identifiers [71,72].

## 4. Conclusions

In summary, the present study demonstrates that cranberry proanthocyanidins significantly modulate cancer-related biological processes and key signaling pathways in NCI-H460 lung cancer cells following a single exposure at a behaviorally achievable concentration. PAC had highly significant and rapid apoptosis inducing effects and potent effects on multiple cell cycle linked genes resulting in decreased cell proliferation and increased cell death. Specifically, PAC increased P21 expression levels, which has been linked to apoptosis resistance; thus, PAC may hold promise as a chemopreventive agent during the early phases of carcinogenesis or may act to re-sensitize cancer cells to apoptosis and chemosensitivity. Further investigation of PACs potential to induce autophagy is also warranted given PACs rapid induction of cell death and up-regulation of p73, a gene linked to both type 1 and type 2 cell death. Mechanistic research on PACs cancer inhibitory potential is ongoing in a larger panel of aerodigestive tract cell lines. 

## References

[1] American Cancer Society (2010). Cancer Facts and Figures 2010.

[2] Blot W.J., Fraumeni J.F., Schottenfeld D., Fraumeni J. (1996). Cancers of the lung and pleura. Cancer Epidemiology and Prevention.

[3] International Agency for Research on Cancer (1987). Overall evaluation of carcinogenicity: An updating of IARC Monographs Volumes 1 to 42. IARC Monogr. Eval. Carcinog. Risks Hum..

[4] World Cancer Research Fund, American Institute for Cancer Research (2007). Food, Nutrition, Physical Activity, and the Prevention of Cancer: A Global Perspective.

[5] Su X., Howell A.B., D'Souza D.H. (2010). Antiviral effects of cranberry juice and cranberry proanthocyanidins on foodborne viral surrogates--a time dependence study *in vitro*. Food Microbiol..

[6] Lipson S.M., Sethi L., Cohen P., Gordon R.E., Tan I.P., Burdowski A., Stotzky G. (2007). Antiviral effects on bacteriophages and rotavirus by cranberry juice. Phytomedicine.

[7] Avorn J., Monane M., Gurwitz J.H., Glynn R.J., Choodnovskiy I., Lipsitz L.A. (1994). Reduction of bacteriuria and pyuria after ingestion of cranberry juice. JAMA.

[8] Foo L.Y., Lu Y., Howell A.B., Vorsa N. (2000). The structure of cranberry proanthocyanidins which inhibit adherence of uropathogenic P-fimbriated *Escherichia coli in vitro*. Phytochemistry.

[9] Foo L., Lu Y., Howell A.B., Vorsa N. (2000). A-type proanthocyanidin trimers from cranberry that inhibit adherence of uropathogenic P-fimbriated Escherichia coli. J. Nat. Prod..

[10] Zhang L., Ma J., Pan K., Go V.L., You W.C. (2005). Efficacy of cranberry juice on *Helicobacter pylori* infection: A double-blind, randomized placebo-controlled trial. Helicobacter.

[11] Howell A.B., Vorsa N., Der Marderosian A., Foo L.Y. (1998). Inhibition of the adherence of P-fimbriated *Escherichia coli* to uroepithelial-cell surfaces by proanthocyanidin extracts from cranberries. N. Engl. J. Med..

[12] Liu M., Lin L.Q., Song B.B., Wang L.F., Zhang C.P., Zhao J.L., Liu J.R. (2009). Cranberry phytochemical extract inhibits SGC-7901 cell growth and human tumor xenografts in Balb/c *nu/nu* mice. J. Agric. Food Chem..

[13] Evans S., Dizeyi N., Abrahamsson P.-A., Persson J. (2009). The effect of a novel botanical agent TBS-101 on invasive prostate cancer in animal models. Anticancer Res..

[14] Ferguson P.J., Kurowska E.M., Freeman D.J. (2006). *In vivo* inhibition of growth of human tumor lines by flavonoid fractions from cranberry extract. Nutr. Cancer..

[15] Hochman N., Houri-Haddad Y., Koblinski J., Wahl L., Roniger M., Bar-Sinai A., Weiss E.I., Hochman J. (2008). Cranberry juice constituents impair lymphoma growth and augment the generation of antilymphoma antibodies in syngeneic mice. Nutr. Cancer.

[16] Prasain J.K., Jones K., Moore R., Barnes S., Leahy M., Roderick R., Juliana M.M., Grubbs C.J. (2008). Effect of cranberry juice concentrate on chemically-induced urinary bladder cancers. Oncol. Rep..

[17] Kresty L.A., Howell A.B., Baird M. (2008). Cranberry proanthocyanidins induce apoptosis and inhibit acid-induced proliferation of human esophageal adenocarcinoma cells. J. Agric. Food Chem..

[18] Slatore C.G., Littman A.J., Hu D.H., Satia J.A., White E. (2008). Long-term use of supplemental multivitamins, vitamin C, vitamin E, and folate does not reduce the risk of lung cancer. Am. J. Respir. Crit. Care Med..

[19] Bjelakovic G., Nikolova D., Gluud L., Simonetti R., Gluud C. (2007). Mortality in randomized trials of antioxidant supplements for primary and secondary prevention: Systematic review and meta-analysis. JAMA.

[20] The Alpha Tocopherol Beta Carotene Cancer Prevention Study Group (1994). The effect of vitamin E and beta carotene on the incidence of lung cancer and other cancers in male smokers. N. Engl. J. Med..

[21] Omenn G., Goodman G., Thornquist M., Cullen M.R., Glass A., Keogh J.P., Meyskens F.I., Valanis B., Williams J.H., Barnhart S., Hammer S. (1996). Effects of a combination of beta carotene and vitamin A on lung cancer and cardiovascular disease. N. Engl. J. Med..

[22] Hoque M.O., Brait M., Rosenbaum E., Poeta M.L., Pal P., Begum S., Dasgupta S., Carvalho A.L., Ahrendt S.A., Westra W.H., Sidransky D. (2010). Genetic and epigenetic analysis of erbB signaling pathway genes in lung cancer. J. Thorac. Oncol..

[23] Memmott R.M., Dennis P.A. (2010). The role of the Akt/mTOR pathway in tobacco carcinogen-induced lung tumorigenesis. Clin. Cancer Res..

[24] Kanehisa M., Goto S., Furumichi M., Tanabe M., and Hirakawa M. (2010). KEGG for representation and analysis of molecular networks involving diseases and drugs. Nucl. Acids Res..

[25] Kanehisa M., Goto S. (2000). KEGG: Kyoto Encyclopedia of Genes and Genomes. Nucl. Acids Res..

[26] Lai S.-L., Pemg R.-P., Hwang J. (2000). p53 gene status modulates the chemosensitivity of non-small cell lung cancer cells. J. Biomed. Sci..

[27] Yang L., Mashima T., Sato S. (2003). Predominate suppression of apoptosome by inhibitor of apoptosis protein in non-small cell ling cancer H460 cells: Therapeutic effect of a novel polyarginine conjugated Smac peptide. Cancer Res..

[28] Chen Z., Naito M., Hori S., Mashima T., Yamori T., Tsuruo T. (1999). A human IAP-family gene, apollon, expressed in human brain cancer cells. Biochem. Biophys. Res. Commun..

[29] Straub C.S. (2011). Targeting IAPs as an Approach to Anti-cancer Therapy. Curr. Top. Med. Chem..

[30] Pore M.M., Hiltermann T.J., Kruyt F.A. (2010). Targeting apoptosis pathways in lung cancer. Cancer Lett..

[31] Jiang Y., Woronicz J.D., Liu W., Goeddel D.V. (1999). Prevention of constitutive TNF receptor 1 signaling by silencer of death domains. Science.

[32] Wang K., Yin X.-M., Chao D.T., Milliman C.L., Korsmeyer S.J. (1996). BID: A novel BH3 domain-only death agonist. Genes Dev..

[33] Kaelin W.G. (1999). The emerging p53 gene family. J. Natl. Cancer Inst..

[34] Bitomsky N., Hofmann T.G. (2009). Apoptosis and autophagy: Regulation of apoptosis by DNA damage signalling - roles of p53, p73 and HIPK2. FEBS J..

[35] Kim K.W., Hwang M., Moretti L., Jaboin J.J., Cha Y.I., Lu B. (2008). Autophagy upregulation by inhibitors of caspase-3 and mTOR enhances radiotherapy in a mouse model of lung cancer. Autophagy.

[36] Nicholson D.W., Ali A., Thornberry N.A., Vaillancourt J.P., Ding C.K., Gallant M., Gareau Y., Griffin P.R., Labelle M., Lazebnik Y.A., Munday N.A., Raju S.M., Smulson M.E., Yamin T.-T., Li V.L., Miller D.K. (1995). Identification and inhibition of the ICE/CED-3 protease necessary for mammalian apoptosis. Nature.

[37] Chipuk J.E., Fisher J.C., Dillon C.P., Kriwacki R.W., Kuwana T., Green D.R. (2008). Mechanism of apoptosis induction by inhibition of the anti-apoptotic BCL-2 proteins. Proc. Natl. Acad. Sci. USA.

[38] Dasgupta P., Kinkade R., Joshi B., Decook C., Haura E., Chellappan S. (2003). Nicotine inhibits apoptosis induced by chemotherapeutic drugs by up-regulating XIAP and survivin. Proc. Natl. Acad. Sci. USA.

[39] Jin Z., Gao F., Flagg T., Deng X. (2004). Nicotine induces multi-site phosphorylation of Bad in association with suppression of apoptosis. J. Biol. Chem..

[40] Xin M., Deng X. (2005). Nicotine inactivation of the proapoptotic function of Bax through phosphorylation. J. Biol. Chem..

[41] Jin Z., Gao F., Flagg T., Deng X. (2004). Tobacco-specific nitrosamine 4-(methylnitrosamino)-1-(3-pyridyl)-1-butanone promotes functional cooperation of Bcl2 and c-Myc through phosphorylation in regulating cell survival and proliferation. J. Biol. Chem..

[42] Zhang H., Holzgreve W., De Geyter C. (2001). Bcl2-L-10, a novel anti-apoptotic member of the Bcl-2 family, blocks apoptosis in the mitochondria death pathway but not in the death receptor pathway. Hum. Mol. Genet..

[43] Liu X., Zou H., Slaughter C., Wang X. (1997). DFF, a heterodimeric protein that functions downstream of caspase-3 to trigger DNA fragmentation during apoptosis. Cell.

[44] Bingle C.D., Craig R.W., Swales B.M., Singleton V., Zhou P., Whyte M.K.B. (2000). Exon skipping in Mcl-1 results in a Bcl-2 homology domain 3 only gene product that promotes cell death. J. Biol. Chem..

[45] Chaudhary P.M., Eby M., Jasmin A., Bookwalter A., Murray J., Hood L. (1997). Death receptor 5, a new member of the TNFR family, and DR4 induce FADD-dependent apoptosis and activate the NF-kappaB pathway. Immunity.

[46] Kitson J., Raven T., Jiang Y.-P., Goeddel D.V., Giles K.M., Pun K.-T., Grinham C.J., Brown R., Farrow S.N. (1996). A death-domain-containing receptor that mediates apoptosis. Nature.

[47] Bowman M.R., Crimmins M.A., Yetz-Aldape J., Kriz R., Kelleher K., Herrmann S. (1994). The cloning of CD70 and its identification as the ligand for CD27. J. Immunol..

[48] Hsu H., Xiong J., Goeddel D.V. (1995). The TNF receptor 1-associated protein TRADD signals cell death and NF-kappa B activation. Cell.

[49] He L., Grammer A.C., Wu X., Lipsky P.E. (2004). TRAF3 forms heterotrimers with TRAF2 and modulates its ability to mediate NF-{kappa}B activation. J. Biol. Chem..

[50] Roth W., Kermer P., Krajewska M., Welsh K., Davis S., Krajewski S., Reed J.C. (2003). Bifunctional apoptosis inhibitor (BAR) protects neurons from diverse cell death pathways. Cell Death Differ..

[51] Boyd J.M., Malstrom S., Subramanian T., Venkatesh L.K., Schaeper U., Elangovan B., D'Sa-Eipper C., Chinnadurai G. (1994). Adenovirus E1B 19 kDa and Bcl-2 proteins interact with a common set of cellular proteins. Cell.

[52] Ohi N., Tokunaga A., Tsunoda H., Nakano K., Haraguchi K., Oda K., Motoyama N., Nakajima T. (1999). A novel adenovirus E1B19K-binding protein B5 inhibits apoptosis induced by Nip3 by forming a heterodimer through the C-terminal hydrophobic region. Cell Death Differ..

[53] Razmara M., Srinivasula S.M., Wang L., Poyet J.-L., Geddes B.J., DiStefano P.S., Bertin J., Alnemri E.S. (2002). CARD-8 protein, a new CARD family member that regulates caspase-1 activation and apoptosis. J. Biol. Chem..

[54] Ahmad M., Srinivasula S.M., Wang L., Talanian R.V., Litwack G., Fernandes-Alnemri T., Alnemri E.S. (1997). CRADD, a novel human apoptotic adaptor molecule for caspase-2, and FasL/tumor necrosis factor receptor-interacting protein RIP. Cancer Res..

[55] Crowe P.D., VanArsdale T.L., Walter B.N., Ware C.F., Hession C., Ehrenfels B., Browning J.L., Din W.S., Goodwin R.G., Smith C.A. (1994). A lymphotoxin-beta-specific receptor. Science.

[56] Rooney I.A., Butrovich K.D., Glass A.A., Borboroglu S., Benedict C.A., Whitbeck J.C., Cohen G.H., Eisenberg R.J., Ware C.F. (2002). The lymphotoxin-beta receptor is necessary and sufficient for LIGHT-mediated apoptosis of tumor cells. J. Biol. Chem..

[57] Cerutti A., Schaffer A., Goodwin R.G., Shah S., Zan H., Ely S., Casali P. (2000). Engagement of CD153 (CD30 ligand) by CD30-positive T cells inhibits class switch DNA recombination and antibody production in human IgD-positive IgM-positive B cells. J. Immun..

[58] Naumovski L., Cleary M.L. (2002). The p53-binding protein 53BP2 also interacts with Bcl2 and impedes cell cycle progression at G2/M. Mol. Cell. Biol..

[59] Samuels-Lev Y., O'Connor D.J., Bergamaschi D., Trigiante G., Hsieh J.-K., Zhong S., Campargue I., Naumovski L., Crook T., Lu X. (2001). ASPP proteins specifically stimulate the apoptotic function of p53. Mol. Cell.

[60] Bendjennat M., Boulaire J., Jascur T., Brickner H., Barbier V., Sarasin H., Fotedar A., Fotedar R. (2003). UV irradiation triggers ubiquitin-dependent degradation of p21(WAF1) to promote DNA repair. Cell.

[61] Wei J., Zhao J., Long M., Han Y., Wang X., Lin F., Ren J., He T., Zhang H. (2010). p21WAF1/CIP1 gene transcriptional activation exerts cell growth inhibition and enhances chemosensitivity to cisplatin in lung carcinoma cell. BMC Cancer.

[62] American Type Culture Collection. NCI-H460, ATCC HTB-177^TM^. http://www.atcc.org/.

[63] Howell A.B., Reed J.D., Krueger C.G., Winterbottom R., Cunningham D.G., Leahy M. (2005). A-type proanthocyanidins and uropathogenic bacterial anti-adhesion activity. Phytochemistry.

[64] Foo L.Y., Lu Y., Howell A.B., Vorsa N. (2000). The structure of cranberry proanthocyanidins which inhibit adherence of uropathogenic P-fimbriated *Escherichia coliin vitro*. Phytochemistry.

[65] Foo L.Y., Lu Y., Howell A.B., Vorsa N. (2000). A-type proanthocyanidin trimers from cranberry that inhibit adherence of uropathogenic P-fimbriated *Escherichia coli*. J. Nat. Prod..

[66] Darzynkiewicz Z., Bruno S., Del Bino G., Gorczyca W., Hotz M.A., Lassota P., Traganos F. (1992). Features of apoptotic cells measured by flow cytometry. Cytometry.

[67] Dennis G., Sherman B.T., Hosack D.A., Yang J., Gao W., Lane H.C., Lempicki R.A. (2003). DAVID: Database for Annotation, Visualization, and Integrated Discovery. Genome Biol..

[68] Auer H., Lyianarachchi S., Newsom D., Klisovic M.I., Marcucci G., Kornacker K. (2003). Chipping away at the chip bias: RNA degradation in microarray analysis. Nat. Genet..

[69] Irizarry R.A., Bolstad B.M., Collin F., Cope L.M., Hobbs B., Speed T.P. (2003). Summaries of Affymetrix Gene Chip probe level data. Nucl. Acids Res..

[70] Raponi M., Zhang Y., Yu J., Chen G., Lee G., Taylor J.M., Macdonald J., Thomas D., Moskaluk C., Wang Y., Beer D.G. (2006). Gene expression signatures for predicting prognosis of squamous cell and adenocarcinomas of the lung. Cancer Res..

[71] Huang D.W., Sherman B.T., Lempicki R.A. (2009). Systematic and integrative analysis of large gene lists using DAVID Bioinformatics Resources. Nat. Protoc..

[72] Thomas P.D., Campbell M.J., Kejariwal A., Mi H., Karlak B., Daverman R., Diemer K., Muruganujan A., Narechania A. (2003). PANTHER: A library of protein families and subfamilies indexed by function. Genome Res..

[73] Mi H., Dong Q., Muruganujan A., Gaudet P., Lewis S., Thomas P.D. (2010). PANTHER version 7: Improved phylogenetic trees, orthologs and collaboration with the Gene Ontology Consortium. Nucl. Acids Res..

